# Supramolecular Hydrogel Showing Mechanical Robustness and Good Adhesion Underwater

**DOI:** 10.1002/advs.202509542

**Published:** 2025-09-15

**Authors:** Xiaohe Zhou, Yihan Cui, Rui Hu, Xiaofan Ji

**Affiliations:** ^1^ Department Key Laboratory of Material Chemistry for Energy Conversion and Storage Ministry of Education Hubei Key Laboratory of Material Chemistry and Service Failure Hubei Engineering Research Center for Biomaterials and Medical Protective Materials State Key Laboratory of Materials Processing and Die and Mould Technology School of Chemistry and Chemical Engineering Huazhong University of Science and Technology Wuhan 430074 P. R. China

**Keywords:** mechanical strength, self‐assembly, supramolecular hydrogel, underwater adhesion

## Abstract

Underwater adhesive materials hold significant application value in fields such as biomedicine and marine engineering. The core performance of these materials lies in the synergistic optimization of their mechanical properties and interfacial adhesive strength. Although supramolecular hydrogels have achieved initial breakthroughs through dynamic bond design, they fail to be mechanically tough and favorably adhesive underwater at the same time. To address this challenge, a strategy to fabricate a supramolecular hydrogel with high crosslinking density using 3D printing technology is proposed. The obtained hydrogel demonstrates excellent mechanical strength due to its high‐density hydrogen bonding crosslinks that it retains a tensile stress of 4 MPa and can support a 20 g load without deformation after submersion in water for 30 min. Meanwhile, it displays remarkable self‐adhesion property underwater, achieving an interfacial adhesion strength of 690 kPa following 20 min of contact duration. Notably, the hydrogel manifests versatile underwater adhesion capabilities to various substrates. Therefore, a supramolecular hydrogel that integrates both good mechanical performance and adhesion underwater characteristics is successfully developed. In addition, modular soft robots are also constructed using this hydrogel, which is able to successfully complete various underwater missions.

## Graphical abstract

In this study, a supramolecular hydrogel integrating mechanical robustness and underwater adhesion is developed through a high‐density supramolecular cross‐linking strategy. The high‐density crosslinking network of the dynamic hydrogen bonds not only endows the material with good mechanical properties but also enables excellent underwater adhesion through water‐induced surface reconstruction with abundant hydrogen‐bonding sites.

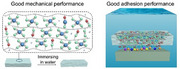

## 1 Introduction

Underwater adhesion, as a crucial link between materials science and engineering applications, holds significant importance across numerous fields.^[^ ^1^, ^2^, ^3^, ^4^, ^5^ ^]^ In marine engineering, underwater adhesives are utilized in watercraft maintenance, including pipeline sealing and sensor fixation.^[^ ^6^, ^7^, ^8^, ^9^, ^10^ ^]^ For biomedical applications, they address the challenge of instant sealing of moist tissues such as myocardium, vascular endothelium, and oral tissues, providing critical technological support for minimally invasive interventions and drug delivery.^[^ ^11^, ^12^, ^13^, ^14^, ^15^ ^]^ In soft robotics and wearable flexible electronics, underwater adhesive materials facilitate the integration of functional devices with biological tissues or aquatic equipment, enabling underwater monitoring and sensing.^[^ ^16^, ^17^, ^18^ ^]^ However, conventional adhesives have shown critical limitations in aquatic environments across these fields.^[^ ^18^, ^19^, ^20^ ^]^ First, hydration layers formed by water molecules at the interface severely hinder contact between adhesive and substrate, resulting in an abrupt decline in adhesive strength underwater. Second, the mechanical properties of the adhesive materials are susceptible to the underwater environment, making it difficult to sustain long‐term adhesive stability under water. These challenges significantly restrict their further development. Consequently, developing underwater adhesives that combine high mechanical strength with strong interfacial bonding remains a critical and pressing challenge in the field of materials science.

The essence of adhesion originates from interfacial non‐covalent interactions.^[^ ^21^, ^22^, ^23^, ^24^, ^25^, ^26^ ^]^ At the same time, supramolecular hydrogels, constructed by assembly of polymer chains via noncovalent interactions, seem a good candidate for an underwater adhesive for their abundant supramolecular units.^[^ ^27^, ^28^, ^29^ ^]^ However, the existing supramolecular hydrogel systems face critical limitations. For example, supramolecular hydrogels based on host‐guest interaction (e.g., cyclodextrin derivatives), despite their water solubility and functional versatility, suffer from low interfacial binding energy due to hydrophobic‐driven molecular recognition, resulting in weak underwater adhesion.^[^ ^30^ ^]^ Other water‐soluble macrocycles (e.g., cucurbiturils, sulfonated calixarenes, and ionic pillararenes) face challenges in hydrogel integration, while neutral macrocycles (e.g., crown ethers, pillararenes) are limited by poor aqueous solubility. Metal‐coordinated hydrogels offer tunable mechanical strength but require specific organic ligands and metal ions, restricting their universality in aquatic environments. Hydrogen bond‐based hydrogel often needs to be combined with hydrophobic interactions to form supramolecular hydrogels, as hydrophobic interactions can protect hydrogen bonds from disruption by water molecules, thus achieving interfacial adhesion.^[^ ^31^ ^]^ However, hydrophobic binding forces are very weak, resulting in weaker mechanical properties for the overall supramolecular hydrogel. Electrostatically driven hydrogels (e.g., quaternary ammonium cation systems) effectively penetrate hydration layers but exhibit insufficient cohesion, failing to meet deep‐sea pressure requirements.^[^ ^32^ ^]^ As a result, the existing supramolecular hydrogels fail to be mechanically tough and favorably adhesive under water at the same time.

**Scheme 1:**
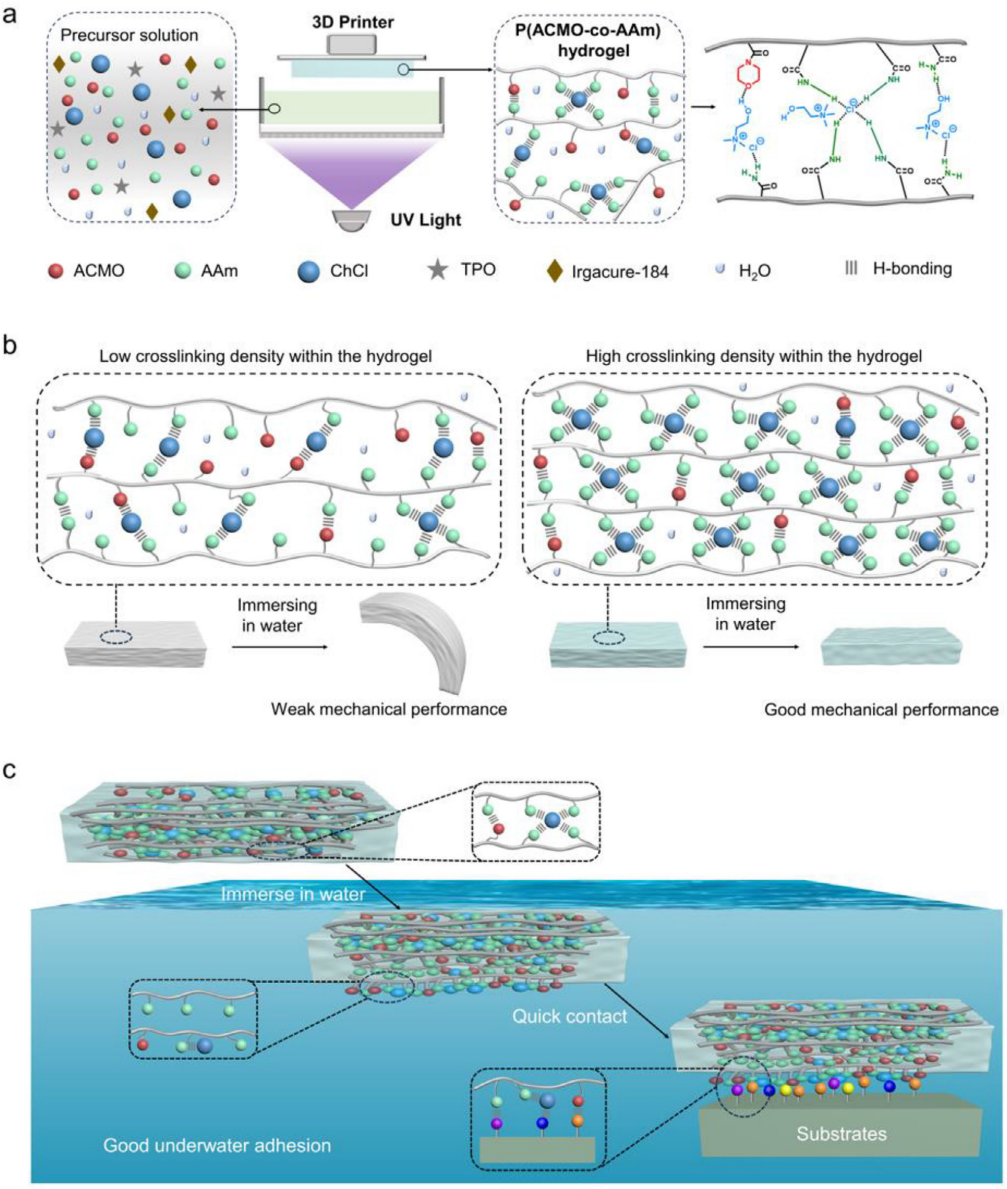
a) Cartoon illustration of the composition of precursor solution, 3D printer, cross‐linked supramolecular polymer network, and chemical structure of hydrogel P(ACMO‐*co*‐AAm). b) Cartoon illustration depicting hydrogel networks with varying crosslinking densities and their changes in mechanical properties after water immersion. c) Schematic diagram of the underwater adhesion mechanism of hydrogel P(ACMO‐*co*‐AAm).

Herein, to address this challenge, we developed a supramolecular hydrogel P(ACMO‐*co*‐AAm) by employing a high‐density crosslinking strategy. The obtained hydrogel was fabricated via 3D printing from a precursor solution containing acrylomorpholine (ACMO), acrylamide (AAm), choline chloride (ChCl), deionized water, diphenyl (2,4,6‐trimethylbenzoyl)phosphine oxide (TPO), and the photo‐initiator Irgacure‐184. The free choline chloride acted as a hydrogen bond acceptor, forming a supramolecular network through hydrogen bonding with amino or carbonyl groups in the polymer chains. Supramolecular hydrogels with varying crosslinking densities were achieved by adjusting the concentration of acrylamide. Lower concentrations resulted in a sparse non‐covalent network, promoting water diffusion into the hydrogel. This led to significant deformation and a sharp decline in mechanical strength upon hydration. In contrast, higher acrylamide levels resulted in a high‐density hydrogen‐bonded network that slowed down the rate of water diffusion, allowing the hydrogels to maintain excellent mechanical properties and rigidity even after water immersion. Simultaneously, the hydrogel exhibited excellent underwater self‐adhesive properties, which were attributed to the dynamic disentanglement of the hydrogen‐bonding network on the hydrogel's surface via water‐mediated competitive dissociation, exposing high‐density hydrogen‐bonding moieties that facilitated strong adhesion through interfacial hydrogen bond reorganization. Furthermore, the hydrogel demonstrated effective underwater adhesion strength to various substrates. Finally, this supramolecular hydrogel was utilized to achieve the underwater assembly of soft robots, and the resulting modularly assembled robot successfully completed various underwater tasks (**Scheme** **1**).

## 2 Results and Discussion

**Figure 1:**
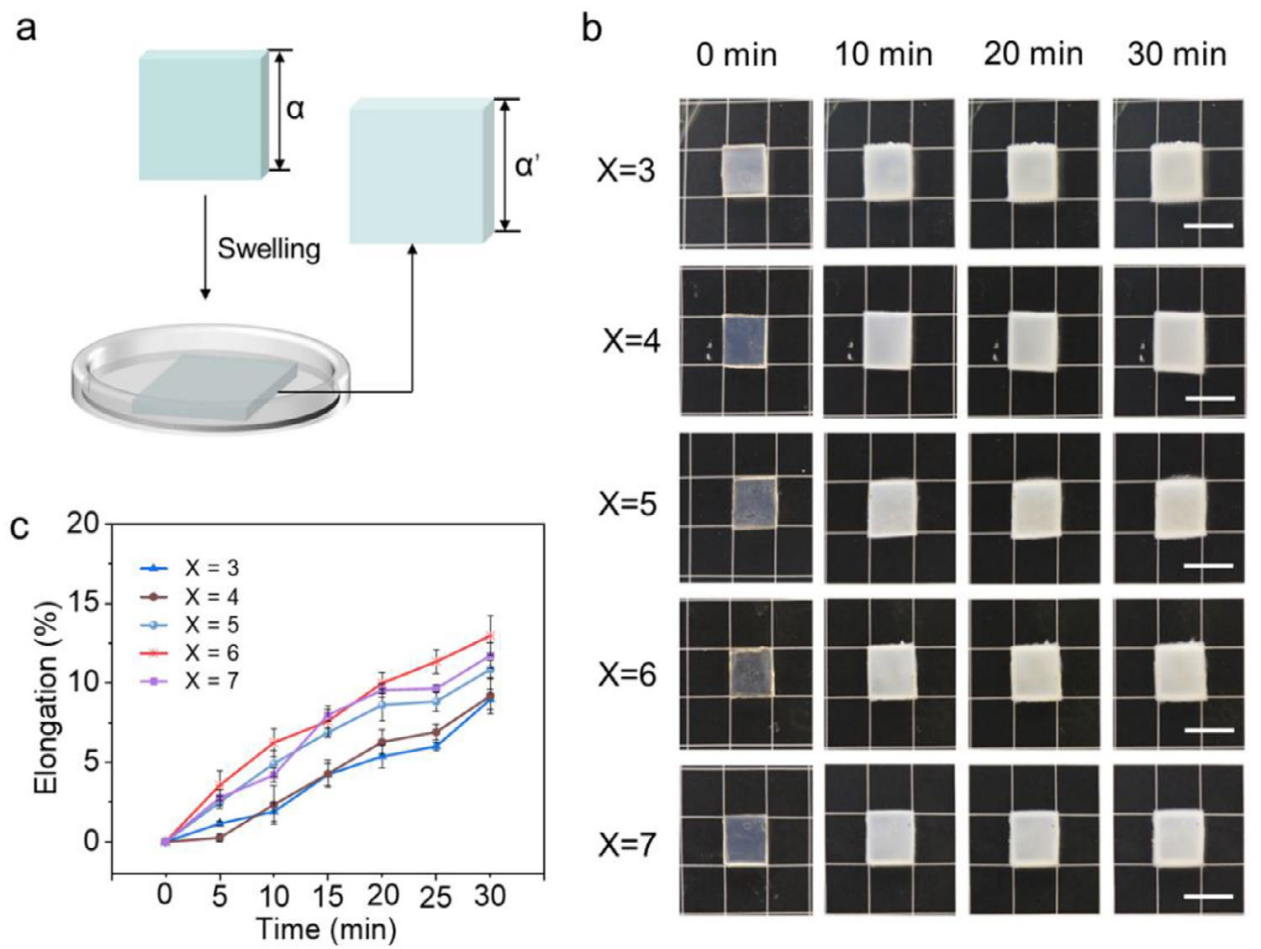
a) Schematic diagram of the swelling experiment. b) Partial photographs indicating hydrogels P(ACMO‐*co*‐AAmx) (*X* = 3, 4, 5, 6, 7) swelling immersed in water for different times (0, 10, 20, and 30 min). The scale bars are 1 cm. c) Plot of size variation versus immersion time.

First, a series of hydrogels P(ACMO‐*co*‐AAm_x_) (*X* = 3, 4, 5, 6, 7) were prepared by 3D printer. More details of the preparation process can be found in the supporting information (SI). Resistance to swelling is a crucial factor in the application of hydrogels in underwater environments. Therefore, swelling behaviors of hydrogels P(ACMO‐*co*‐AAm_x_) (*X* = 3–7) with different ratios were investigated (**Figure** **1**a). As shown in Figure 1b,c, the size change rate of hydrogel samples did not exceed 15% after 30 min of immersion in water when *X* = 3–7. All these samples did not change much in size after immersion in water.

**Figure 2:**
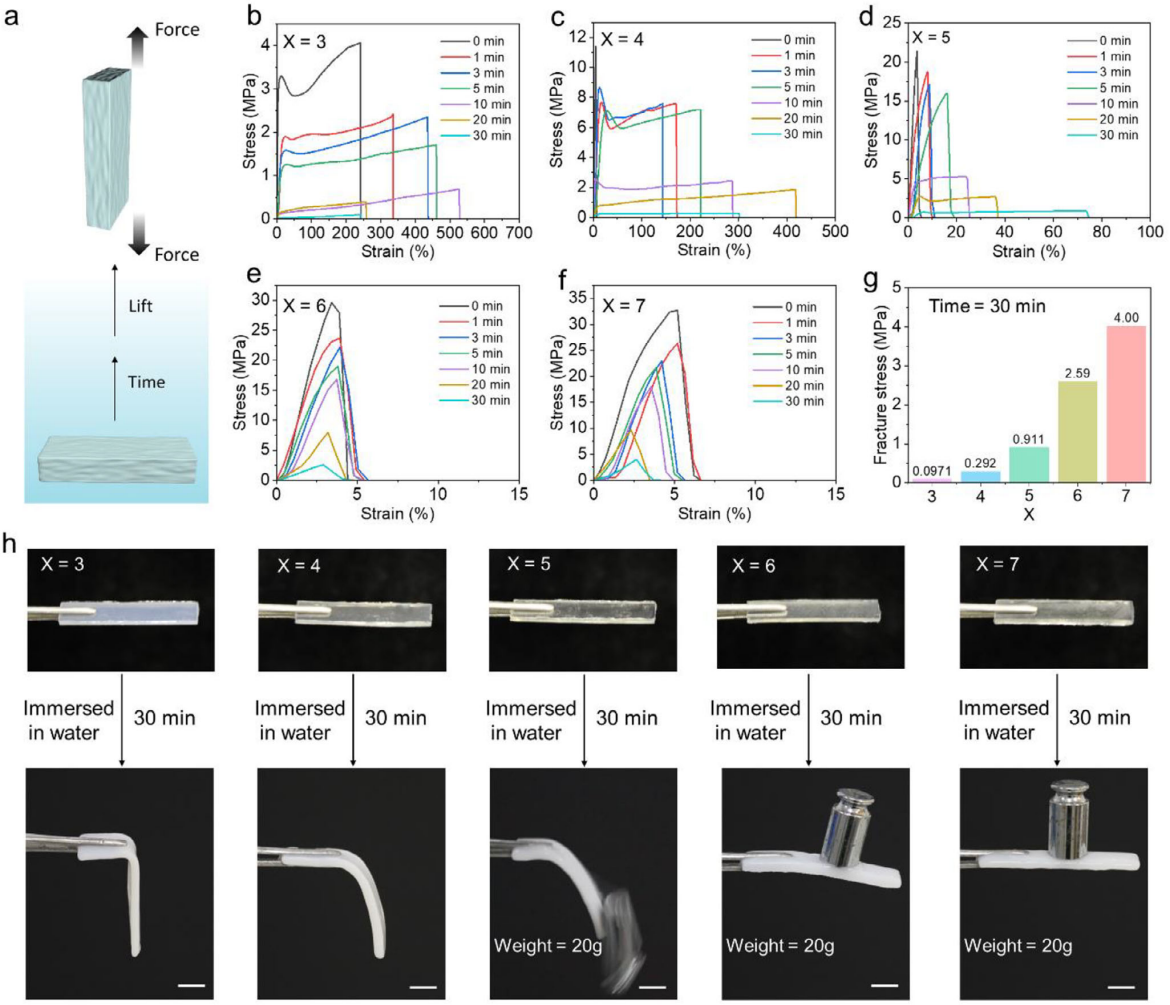
a) Schematic diagram of tensile test. b–f) Stress–strain curves of hydrogels P (ACMO‐*co*‐AAm_x_) (*X* = 3, 4, 5, 6, 7) after immersion in water for various durations (0, 1, 3, 5, 10, 20, and 30 min). g) Histogram of fracture stress after 30 min of immersion of hydrogels P(ACMO‐*co*‐AAm_x_) (*X* = 3, 4, 5, 6, 7). h) Photographs illustrating the load‐bearing capacity of hydrogels with varying acrylamide content after water immersion for 30 min. The scale bars are 1 cm.

Subsequently, in order to investigate their mechanical properties, tensile tests were carried out on hydrogel s P(ACMO‐*co*‐AAm_x_). First, the hydrogels P(ACMO‐*co*‐AAm_x_) with different acrylamide contents (*X* = 3, 4, 5, 6, 7) were immersed in water for different times (0, 1, 3, 5, 10, 20, and 30 min), respectively, which were followed by tensile tests (**Figure** **2**a). As shown in Figure 2b, When *X* = 3, the fracture stress of the hydrogel P(ACMO‐*co*‐AAm_3_) decreased from 4.06 to 0.0971 MPa as the immersion time in water increased from 0 to 30 min. The fracture strain initially increased from 240% to 526%, then decreased to 238%. When *X* = 4, the fracture stress reduced from 11.4 to 0.292 MPa, and the strain increased from 5.47% to 417%, then decreased to 300% (Figure 2c). For *X* = 5, the stress decreased from 21.4 to 0.911 MPa, while the strain increased from 7% to 78% (Figure 2d). When *X* = 6 and 7, the fracture stress declined from 29.8 and 32.4 MPa, respectively to 2.59  and 4.00 MPa, with relatively minor changes in strain, which remained around 6% (Figure 2e,f). Meanwhile, we compared the mechanical strength of hydrogels containing different proportions of acrylamide after immersion in water for the same duration (Figure 2g). The results indicated that at *X* = 6 and 7, the hydrogels still exhibited relatively good mechanical strength after immersion, with stress values reaching 2.59 and 4.00 MPa, respectively. These findings indicated that increasing the acrylamide content promoted the formation of a high‐density 3D network through non‐covalent interactions (hydrogen bonding), which in turn slowed down the diffusion of water molecules into the hydrogel network. Consequently, the hydrogel material maintained relatively good mechanical strength even after soaking in water for a certain period. Furthermore, we visually demonstrated the mechanical strength of the hydrogels after water immersion through load‐bearing experiments. As shown in the Figure 2h, when *X* = 3 and 4, the hydrogels became very soft after water immersion, lacking sufficient support strength to hold weights. When *X* = 5, the hydrogel still possessed some mechanical integrity but was unable to support a 20 g weight. Conversely, when *X* = 6 and 7, the hydrogels maintained good rigidity and mechanical strength after 30 min of water immersion, capable of supporting a 20 g weight. These results indicate that high‐crosslink‐density hydrogels (*X* = 6 and 7) retain considerable mechanical strength even after immersion in water. Therefore, hydrogels with *X* = 6 and 7 were selected for further detailed characterization and testing. Additionally, we investigated the effect of varying choline chloride content on the properties of the hydrogel. As shown in Figure S2 (Supporting Information) with an increase in choline chloride concentration, the hydrogel's deformability was significantly enhanced, with tensile strains reaching up to 600%. However, its tensile strength remained at only 4 MPa. Furthermore, after soaking in water for 30 min, its mechanical performance deteriorated sharply, with the tensile strength decreasing to 0.02 MPa (Figure S3, Supporting Information). Therefore, excessively high choline chloride content may not be suitable for underwater environments.

**Figure 3:**
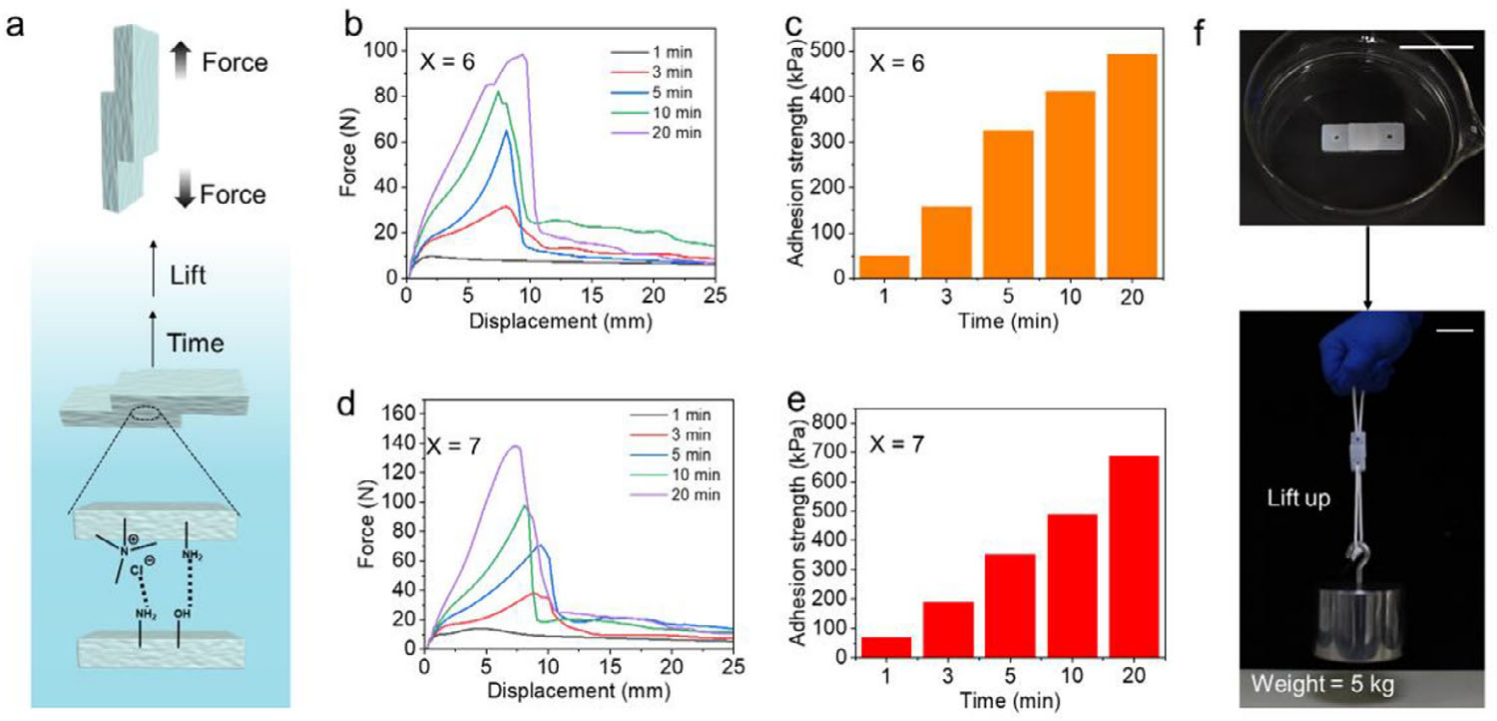
a) Schematic diagram of the lap shear tensile test. b–e) Force‐displacement curves and histogram of adhesion strength versus adhesion time for hydrogels P(ACMO‐*co*‐AAm_6_) and P(ACMO‐*co*‐AAm_7_) adhered underwater for different durations. f) The photograph of two adhered hydrogels lifting a 5 kg weight. The scale bars are 5 cm.

To study the adhesion property, lap shear tensile tests were conducted on hydrogel P(ACMO‐*co*‐AAm_6_) and hydrogel P(ACMO‐*co*‐AAm_7_) (**Figure** **3**a). Due to the initial hydrogels P(ACMO‐co‐AAm_6_) and P(ACMO‐co‐AAm_7_) being too rigid with poor surface fluidity and exhibiting almost no adhesiveness in air (Figure S4, Supporting Information), adhesion tests were not performed on its initial state. First, the hydrogel samples of P(ACMO‐*co*‐AAm_6_) were subjected to adhesion under water for different durations (1, 3, 5, 10, and 20 min), after which shear tensile tests were conducted on each sample. For the hydrogel P(ACMO‐co‐AAm_6_), the force‐displacement curves indicated that, as the adhesion time in water increased from 1 to 20 min, the maximum fracture force increased from 9.59 to 98.5 N, and the adhesion strength rose from 48.5 to 493 kPa (Figure 3b,c). Similarly, P(ACMO‐*co*‐AAm_7_) demonstrated maximum fracture force improvement from 13.9 to 138 N with corresponding adhesion strength increased from 69.6 to 690 kPa under identical conditions (Figure 3d,e). Furthermore, lap shear tensile tests were also performed on hydrogel P(ACMO‐*co*‐AAm_6_) and hydrogel P(ACMO‐*co*‐AAm_7_) after 30 min of water immersion. However, both samples fractured at the non‐adhesive regions, thereby precluding direct measurement of the adhesive strength. This phenomenon was attributed to the prolonged underwater adhesion, which resulted in an interfacial adhesion strength exceeding the hydrogel's cohesive strength, leading to bulk hydrogel fracture (Figure S5, Supporting Information). As a result, hydrogel P(ACMO‐*co*‐AAm_7_) demonstrated good adhesion properties underwater, and the adhesion strength was time‐dependent. The good underwater adhesion of hydrogels originated from water‐induced disruption of surface hydrogen‐bonding networks, which exposes high‐density hydrogen‐bonding moieties to facilitate interfacial interactions. Simultaneously, water‐mediated enhancement of interfacial fluidity promoted polymer chain migration and interdiffusion across the interfaces, synergistically strengthening adhesive performance.^[^ ^33^ ^]^ Benefitting from their excellent underwater adhesion, a weight of as high as 5 kg can be steadily hung up through the two adhered hydrogels P(ACMO‐*co*‐AAm_7_) (Figure 3f).

**Figure 4:**
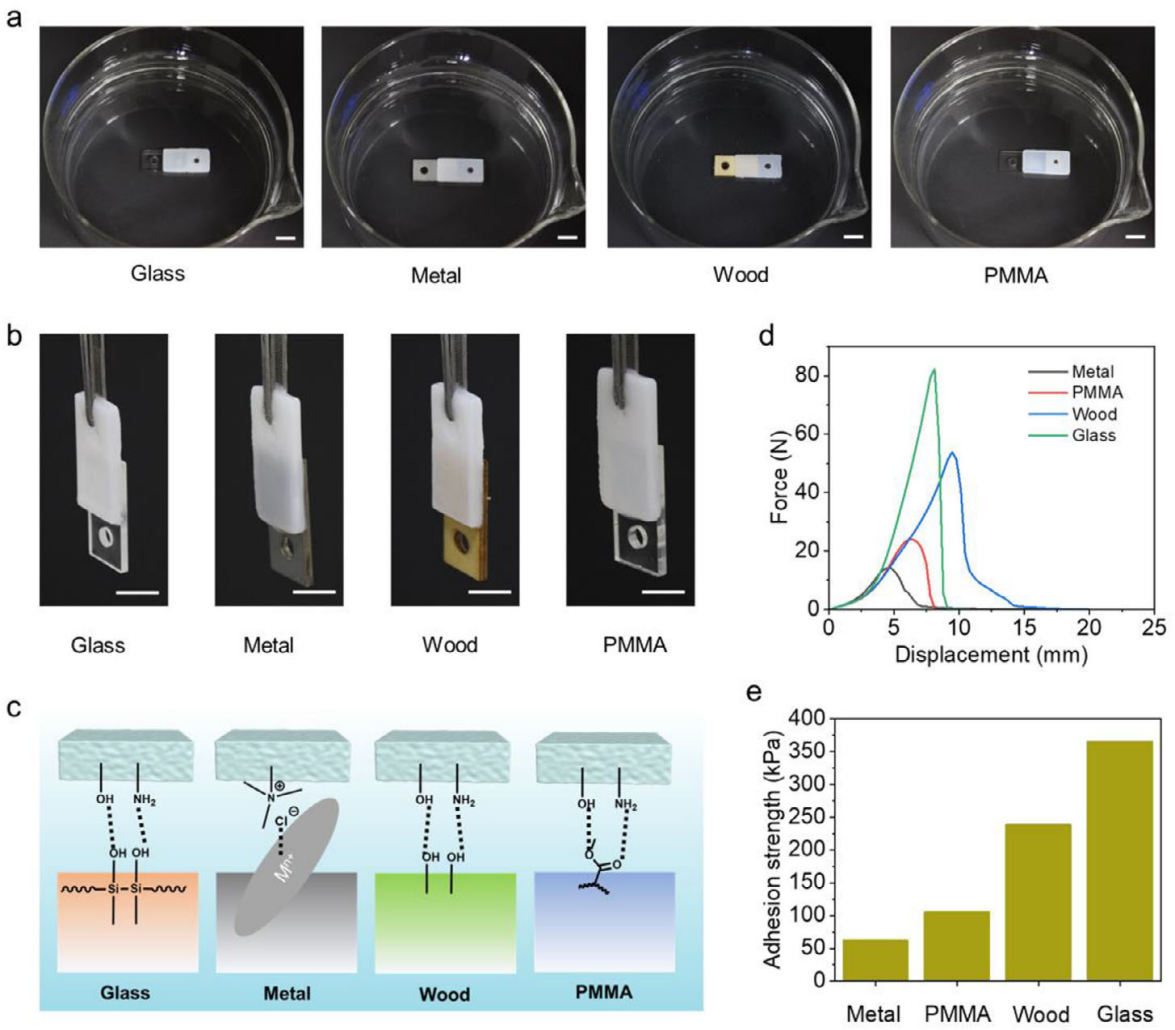
a,b) Photographs of hydrogel P(ACMO‐*co*‐AAm_7_) adhering to various substrates underwater, as well as images of the samples after removal from the water surface. The scale bars are 1 cm. c) Adhesion mechanism between hydrogels and different substrates. d,e) Force‐displacement curves and histogram of adhesion strength for hydrogel P(ACMO‐*co*‐AAm_7_).

Due to the exposure of numerous hydrogen bonding groups on the surface of the hydrogel when submerged under water, it can serve as an effective underwater adhesive. Subsequently, to verify the universality of underwater adhesion of this hydrogel, lap shear tensile experiments were performed between the hydrogel and various substrates (including metal, wood, glass, and PMMA) (**Figure** **4**a–c). As Figure 4d,e shows, the force‐displacement curves revealed that the maximum fracture forces generated between the hydrogel and glass, metal, PMMA, and wood were 14.1, 23.9, 53.8, and 82.2 N, respectively, with corresponding adhesion strengths of 62.8, 106, 239, and 366 kPa. The good underwater adhesion of this hydrogel across diverse substrates could be attributed to the water‐induced generation of abundant hydrogen‐bonding groups on its surface, which dynamically interacted with the interfacial layers of the substrates. To further evaluate the supramolecular hydrogel developed in this work, the corresponding comparison of other supramolecular adhesive hydrogels is shown in **Table** **1**. The hydrogel exhibited good adhesion and mechanical robustness to most of the common hydrogel adhesives.

**Table 1:** Comparison of other adhesive supramolecular hydrogels.

Adhesive hydrogel	Adhesion mechanics	Substrate	Underwater adhesion strength [kPa]	Tensile strength [MPa]	References
PBA‐Aa‐AA hydrogel	Hydrogen bonds	Glass	40	0.2	[34]
PVA hydrogel	Hydrogen bonds	Glass	305	/	[35]
MXene/PHMP hydrogel	Hydrogen bonds	Glass	17	0.1	[36]
Poly(cation‐adj‐π) hydrogel	Electrostatic interactions	Glass	60	0.4	[32]
PSA‐DA/DES hydrogel	Hydrogen bonds and electrostatic interactions	Glass	50	/	[31]
P(ACMO‐co‐AAm) hydrogel	Hydrogen bonds	Glass	366	4	This work

In order to study the cyclic adhesion stability of hydrogel P(ACMO‐*co*‐AAm_7_), repeated adhesion tests were conducted under water (Figure S6 and Movie S1, Supporting Information). First, two P(ACMO‐*co*‐AAm_7_) hydrogel modules of the same size were placed in water, which were overlapped mutually at the end and formed a short‐chain hydrogel assembly via interfaced adhesion given by hydrogen bonds between hydrogel module interfaces. After successful assembly, the short‐chain hydrogel assembly would not drop off even if shaken violently in water, showing favorable stability underwater. Next, the hydrogel assembly was taken out of the water and disassembled through external force. Subsequently, two separated hydrogel modules were placed in water again and contacted with each other, for which reassembly was completed via interface adhesion. Following secondary assembly, violent shaking was imposed for a second time on the hydrogel assembly, but it still failed to disassemble it. It was observed that the secondary hydrogel assembly maintained its structural integrity after being taken out of the water. It was thus shown that hydrogel P(ACMO‐*co*‐AAm_7_) had repeatable adhesion ability in water.

**Figure 5:**
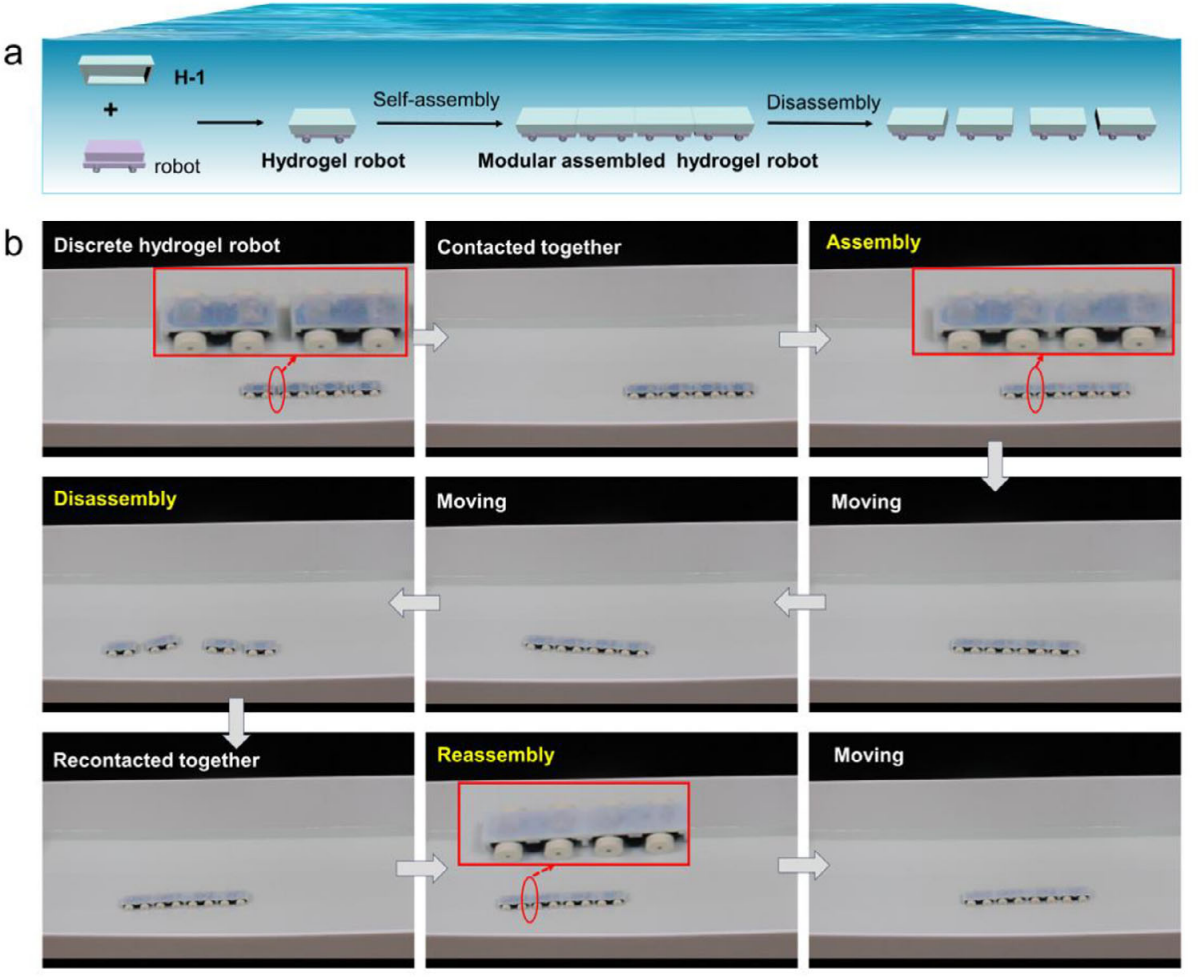
a) Cartoon schematic diagrams of the assembly and disassembly of hydrogel robots. b) Photographs indicating assembly, disassembly, and reassembly processes of the **MAHR**. The length of a single hydrogel robot was 11 cm.

Based on favorable mechanical property and underwater adhesion, hydrogel P(ACMO‐*co*‐AAm_7_) was employed in constructing an underwater soft robot. To further investigate the underwater assembly behavior of hydrogel robots, we combined the hydrogel module **H‐1** with the robot in assembly to obtain a hydrogel robot (**Figure** **5**a). Initially, four hydrogel robots are discretely placed on the right side of the underwater platform (Figure 5b; Movie S2, Supporting Information). By remotely controlling the movement, the four hydrogel robots were connected to each other bumper‐to‐bumper through hydrogen bonds between hydrogel interfaces, thus completing the assembly of a linear modular assembled hydrogel robot (**MAHR**) with a larger size. After successful assembly, the linear **MAHR** was able to move stably underwater without any disassembly behavior when controlling and unifying its movement direction at the same time. By simultaneously controlling the hydrogel robots at different positions to move in opposite directions subsequently, the linear **MAHR** performed disassembly behavior and reformed into four discrete underwater robots again based on the reversible adhesion of the hydrogel. Finally, the disassembled four hydrogel robots were brought back into contact and reassembled through interfacial adhesion to form the linear **MAHR** again, which was able to move under water stably by unifying its movement direction. As a result, the **MAHR** could easily achieve the assembly‐disassembly behaviors under water based on reversible interfacial adhesion of the hydrogel. Besides, the hydrogel robot completed the reassembly process due to repetitive adhesion ability through interface hydrogen bonds, which performed stable movement underwater.

**Figure 6:**
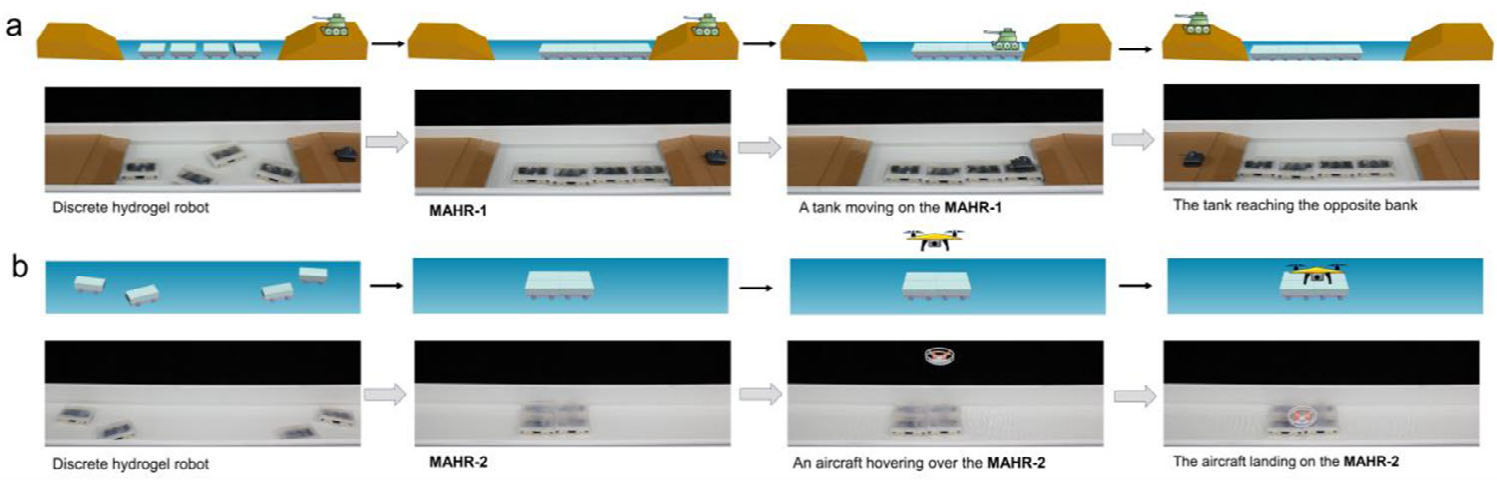
a) Cartoon illustration and photographs of a tank crossing the river supported by **MAHR‐1**. b) Cartoon illustration and photographs of aircraft landing above water supported by **MAHR‐2**.

This adhesive hydrogel exhibited application potential in modular assembled hydrogel robots. For instance, when a tank needed to cross the river, a linear **MAHR‐1** was constructed as a movable bridge via self‐assembly of robots based on this adhesive hydrogel in water (**Figure** **6**a; Movie S3, Supporting Information). In that case, the tank could travel on the bridge and further be transported to the opposite side of the river. Moreover, when an aircraft needed to land on the sea, a flat **MAHR‐2** was built as a parking apron via self‐assembly of robots based on this adhesive hydrogel in water. In this way, the aircraft was able to land on the parking apron steadily (Figure 6b; Movie S4, Supporting Information). Besides the potential for military application, the modular robot based on this adhesive hydrogel could also be employed in underwater waste removal and rescue tasks (Figures S7 and S8 and Movies S5 and S6, Supporting Information).

## 3 Conclusion

In summary, we successfully developed a supramolecular hydrogel integrating mechanical robustness and underwater adhesion through a high‐density supramolecular cross‐linking strategy. The high density of the dynamic hydrogen‐bonding crosslinking network not only endowed the material with good mechanical properties, maintaining a tensile strength of 4 MPa after 30‐minute water immersion, but also enabled good underwater adhesion through water‐induced surface reconstruction with abundant hydrogen‐bonding sites. The maximum adhesion strength progressively increased to 690 kPa with prolonged adhesion time. Meanwhile, it can also exhibit favorable underwater adhesion performance with various other substrates. Furthermore, this supramolecular hydrogel demonstrated successful application in the reversible assembly of underwater soft robots and operation in complex scenarios. This study not only established a novel strategy for constructing supramolecular hydrogels that synergistically combined mechanical performance and underwater adhesion, but also contributed to the advancement of underwater adhesive materials. Moreover, it paved the way for future designs of intelligent and durable underwater adhesives with expanded functionalities.

## Conflict of Interest

The authors declare no conflict of interest.

## Author Contributions

X.J. conceived and designed the study. X.Z. and R.H. wrote the paper. X.Z. and Y.C. performed the experiments. X.Z. analyzed the data. X.J., X.Z. and R.H. revised the paper. All authors read and approved the final manuscript.

## Supporting information



Supporting Information

Supplemental Movie 1

Supplemental Movie 2

Supplemental Movie 3

Supplemental Movie 4

Supplemental Movie 5

Supplemental Movie 6

## Data Availability

The data that support the findings of this study are available in the supplementary material of this article.

## References

[1] H. Fan , J. P. Gong , Adv. Mater. 2021, 33, 2102983. 10.1002/adma.202102983 34532910

[2] S. Gao , J. Chen , Y. Zheng , A. Wang , D. Dong , Y. Zhu , Y. Zhang , W. Fang , J. Jin , Adv. Funct. Mater. 2022, 32, 2205990.

[3] L. Yan , X. Yang , H. Zeng , Y. Zhao , Y. Li , X. He , J. Ma , L. Shao , J. Membr. Sci. 2023, 668, 121243.

[4] S. J. Wu , X. Zhao , Chem. Rev. 2023, 123, 14084. 37972301 10.1021/acs.chemrev.3c00380

[5] J. Wei , P. Xiao , T. Chen , Adv. Mater. 2023, 35, 2211758. 10.1002/adma.202211758 36857417

[6] X. Li , Y. Deng , J. Lai , G. Zhao , S. Dong , J. Am. Chem. Soc. 2020, 142, 5371. 32092262 10.1021/jacs.0c00520

[7] S. Y. Zheng , J. Zhou , S. Wang , Y. J. Wang , S. Liu , G. Du , D. Zhang , J. Fu , J. Lin , Z. L. Wu , Q. Zheng , J. Yang , Adv. Funct. Mater. 2022, 32, 2205597.

[8] Z. Tang , X. Lin , M. Yu , A. K. Mondal , L. Huang , L. Chen , H. Wu , ACS Appl. Polym. Mater. 2024, 6, 806.

[9] S. Li , P. Sun , W. Dou , W. Ji , Q. Wang , X. Li , C. Liu , J. Zhao , H. Liu , H. Fan , X. Hou , X. Yuan , Chem. Eng. J. 2024, 479, 147639.

[10] A. Farooq , H. Wanyan , Q. Li , S. Lu , W. Huang , M. Waqas , B. Hong , L. Huang , L. Chen , X. Zhou , H. Wu , Carbohydr. Polym. 2025, 358, 123534. 40383592 10.1016/j.carbpol.2025.123534

[11] Y. Zhao , S. Song , X. Ren , J. Zhang , Q. Lin , Y. Zhao , Chem. Rev. 2022, 122, 5604. 35023737 10.1021/acs.chemrev.1c00815

[12] S. Khattak , I. Ullah , M. Sohail , M. U. Akbar , M. A. Rauf , S. Ullah , J. Shen , H. T. Xu , Aggregate 2024, 6, 688.

[13] S. Balakrishnan , R. Remesh , K. K. Kalathil , A. Y. , Supramol. Mater. 2025, 4,100081.

[14] M. Li , H. Lu , M. Pi , H. Zhou , Y. Wang , B. Yan , W. Cui , R. Ran , Adv. Sci. 2023, 10, 2304780. 10.1002/advs.202304780 PMC10646223 37750254

[15] M. Li , H. Qu , Q. Li , S. Lu , Y. Wu , Z. Tang , X. Liu , Z. Yuan , L. Huang , L. Chen , H. Wu , Chem. Eng. J. 2024, 498, 155552.

[16] S. Li , P. Xiao , Q. Wang , J. He , X. Liu , J. Wei , Y. Wang , T. Chen , ACS Nano 2024, 18, 20694. 10.1021/acsnano.4c06714 39051442

[17] J. Hu , Y. Liu , C. Yang , S. Wu , H. Wang , Y. Qin , Y. Yong , L. Liu , X. Li , S. Gu , Y. Hu , P. Li , J. Huang , Q. Zhang , M. Pan , Adv. Funct. Mater. 2024, 35, 2418681.

[18] A. Narayanan , A. Dhinojwala , A. Joy , Chem. Soc. Rev. 2021, 50, 13321. 34751690 10.1039/d1cs00316j

[19] X. Ma , X. Zhou , J. Ding , B. Huang , P. Wang , Y. Zhao , Q. Mu , S. Zhang , C. Ren , W. Xu , J. Mater. Chem. A 2022, 10, 11823.

[20] S. Wang , J. Liu , L. Wang , H. Cai , Q. Wang , W. Wang , J. Shao , X. Dong , Adv. Mater. Technol. 2022, 8, 2201477.

[21] X. Ji , M. Ahmed , L. Long , N. M. Khashab , F. Huang , J. L. Sessler , Chem. Soc. Rev. 2019, 48, 2682. 31012443 10.1039/c8cs00955d

[22] D. Xia , P. Wang , X. Ji , N. M. Khashab , J. L. Sessler , F. Huang , Chem. Rev. 2020, 120, 6070. 32426970 10.1021/acs.chemrev.9b00839

[23] Z. Li , Y. Han , F. Nie , M. Liu , H. Zhong , F. Wang , Angew. Chem., Int. Ed. 2021, 60, 8212. 10.1002/anie.202015846 33450117

[24] Y. Liu , L. Wang , L. Zhao , Y. Zhang , Z. T. Li , F. Huang , Chem. Soc. Rev. 2024, 53, 1592. 38167687 10.1039/d3cs00705g

[25] J. Liu , Y. S. Huang , Y. Liu , D. Zhang , K. Koynov , H. J. Butt , S. Wu , Nat. Chem. 2024, 16, 1024. 38459235 10.1038/s41557-024-01476-2 PMC11164683

[26] H. Liu , R. Hu , Z.‐Q. Hu , X.‐F. Ji , Chinese J. Polym. Sci. 2024, 42, 1403.

[27] A. H. Hofman , I. A. van Hees , J. Yang , M. Kamperman , Adv. Mater. 2018, 30, 1704640. 10.1002/adma.201704640 29356146

[28] W. Guan , W. Jiang , X. Deng , W. Tao , J. Tang , Y. Li , J. Peng , C. L. Chen , K. Liu , Y. Fang , Angew. Chem., Int. Ed. 2023, 62, 202303506. 10.1002/anie.202303506 37016787

[29] Y. Tian , L. X. Hou , X. N. Zhang , M. Du , Q. Zheng , Z. L. Wu , Small 2024, 20, 2308570. 10.1002/smll.202308570 38716740

[30] A. Harada , R. Kobayashi , Y. Takashima , A. Hashidzume , H. Yamaguchi , Nat. Chem. 2010, 3, 34. 21160514 10.1038/nchem.893

[31] Z. Zhang , A. Yao , P. Raffa , Adv. Funct. Mater. 2024, 34,2407529.

[32] H. Fan , J. Wang , Z. Tao , J. Huang , P. Rao , T. Kurokawa , J. P. Gong , Nat. Commun. 2019, 10, 5127. 31719537 10.1038/s41467-019-13171-9 PMC6851134

[33] Y. Li , R. K. Kankala , L. Wu , A.‐Z. Chen , S.‐B. Wang , ACS Appl. Polym. Mater. 2023, 5, 991.

[34] X. Liu , Q. Zhang , L. Duan , G. Gao , ACS Appl. Mater. Interfaces 2019, 11, 6644. 30666868 10.1021/acsami.8b21686

[35] L. Xu , S. Gao , Q. Guo , C. Wang , Y. Qiao , D. Qiu , Adv. Mater. 2020,32, 2004579. 10.1002/adma.202004579 33169449

[36] S. He , B. Guo , X. Sun , M. Shi , H. Zhang , F. Yao , H. Sun , J. Li , ACS Appl. Mater. Interfaces 2022, 14, 45869. 36165460 10.1021/acsami.2c13371

