# Supplementary material for: Supramolecular Hydrogel Showing Mechanical Robustness and Good Adhesion Underwater
Source: Adv Sci (Weinh). 2025 Sep 15;12(40):e09542. doi: 10.1002/advs.202509542 (PMC12561207; doi:10.1002/advs.202509542)
Supplement: Supplementary file 1 — Supporting Information [file ADVS-12-e09542-s003.docx]

Supporting Information

Supramolecular Hydrogel Showing Mechanical robustness and Good Adhesion Underwater

Xiaohe Zhou, Yihan Cui, Rui Hu and Xiaofan Ji*

Key Laboratory of Material Chemistry for Energy Conversion and Storage, Ministry of Education, Hubei Key Laboratory of Material Chemistry and Service Failure, Hubei Engineering Research Center for Biomaterials and Medical Protective Materials, State Key Laboratory of Materials Processing and Die & Mould Technology, School of Chemistry and Chemical Engineering, Huazhong University of Science and Technology
Wuhan 430074, (P.R. China)

E-mail: xiaofanji@hust.edu.cn

**Materials and Method**

All reagents and solvents were purchased from commercial suppliers and used as received. The hydrogel modules were prepared using an LCD printer (CT-005PRO, CREALTY, China). The target-printed pattern was designed in 3D MAX and sliced for 3D images by HALOT BOX software. The tensile tests of hydrogel were investigated using an electronic universal testing machine (CMT4104, Shenzhen San Testing Machine Co.) with a tensile rate of 200 mm min^-1^. The commercially customized robot used in the experiments measured 11 × 8 × 4 cm (length × width × height), weighed 220 g, and was designed for application in the study.

**3D Printing**

An LCD printer (CT-005PRO, CREALTY, China) was employed to fabricate hydrogel samples and robot shell using precursor solution. The light intensity of the LCD printer was 1.0 mW/cm^2^. The thickness of each slicing layer was set as 0.05 mm. The exposure time of 3D printing for all layers was 1.8 s/layer. After printing, the samples were washed with ethanol and irradiated with a UV lamp for approximately 30 seconds to remove residual liquid resin from their surfaces.

**Fabrication of adhesive hydrogels**

Acrylamide (AAm, monermer), 4-acryloylmorpholine (ACMO, 100 mL), and choline chloride (ChCl, 36.25 g, 260 mmol) were mixed. The mixtures were then heated to 70 °C and stirred for 5 h to form homogeneous colorless solutions. Subsequently, photoinitiator triphenyl phosphite (TPO, 2.57 g, 8.28 mmol) and 1-hydroxycyclohexyl phenyl ketone (Irgacure 184, 2.57 g, 12.6 mmol) were added to the mixtures. The mixtures were incubated for another 30 min in the dark to ensure uniform mixing. The precursor solution was obtained by mixing the mixtures and deionized water in volume ratio 6:1. The hydrogel can be obtained by photocuring a precursor solution using the LCD printer. The prepared hydrogel is denoted as P(ACMO-co-AAm_X_), where X represents the molar ratio of the "AAm" monomer to the "ChCl" monomer (X = n_AAm_ / n_ChCl_).

**Fabrication of hydrogel robots**

The hydrogel robot was prepared by two-step. First, the soft shell of the robot was prepared hydrogel which can be obtained by 3D printer. Next, by combining hydrogels with specific shaped shells with robots, hydrogel robots could be created.

**Swelling Tests**

Test samples 10 mm in length, 10 mm in width, and 2 mm in thickness) were used for the swelling tests. Test samples were individually immersed in water for durations of 0, 5, 10, 15, 20, 25, and 30 minutes, respectively. Following immersion, the edge length dimensions of each sample were measured. (Initial dimensions=α; Swollen dimensions=α’)

Elongation (%) = (α’-α)/α*100%

**Tensile Tests**

Test samples (50 mm in length, 10 mm in width, and 2 mm in thickness) were used for the uniaxial tensile test at a testing velocity of 200 mm min^-1^ in air.

**Adhesion Tests**

The adhesion abilities were evaluated by lap-shear test using the universal tension machine at a speed of 200 mm·min^-1^ in the air. The sample was cut into a 50 × 10 mm^2^ square with a thickness of 2 mm. The adhesion strength (τs) was defined as the maximum tensile force (F_max_) per nominal contact area as τs = F_max_/(wl), where w and l were the width and length of the contact area, respectively (w = 10 mm, l = 20mm).


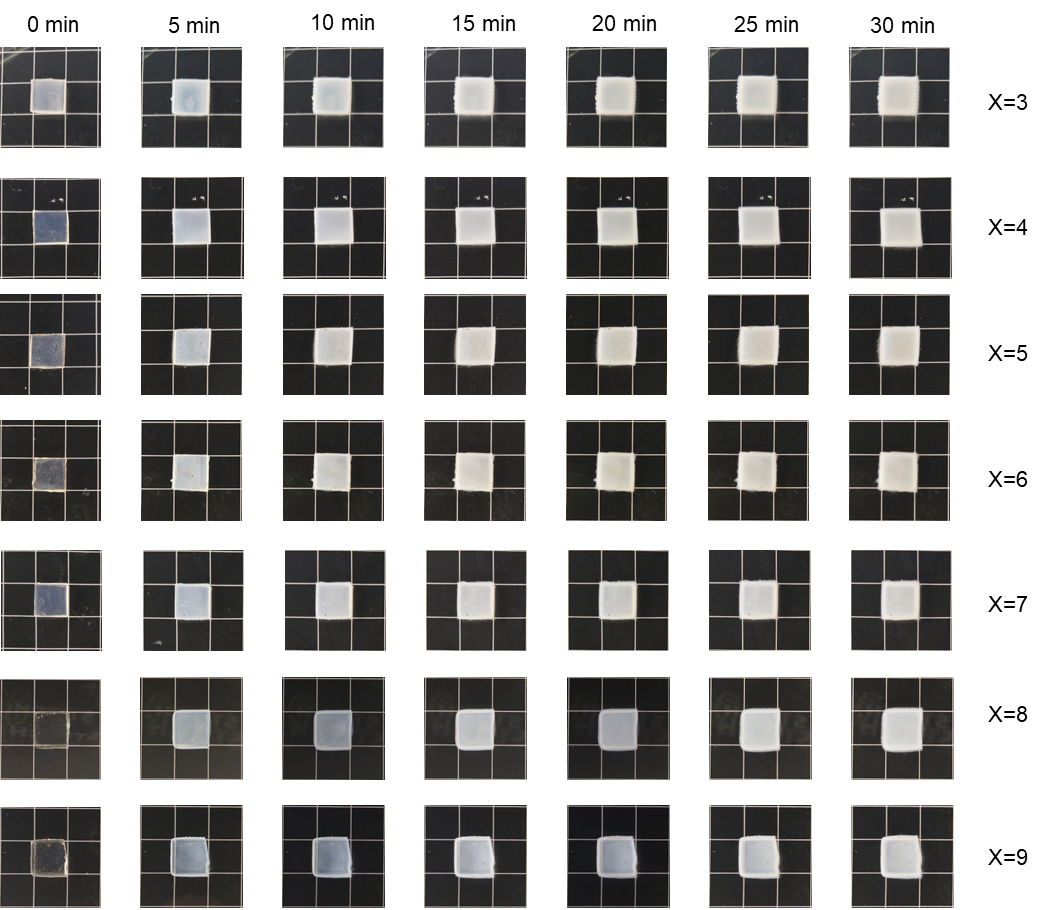


**Figure S1.** Photographs of hydrogels P(ACMO-*co*-AAm_x_) (X = 3, 4, 5, 6, 7) with different acrylamide contents soaked underwater for different durations.

**Figure S2.** Stress-strain curves of hydrogel P (ACMO-*co*-AAm_7_) with varying ChCl content.


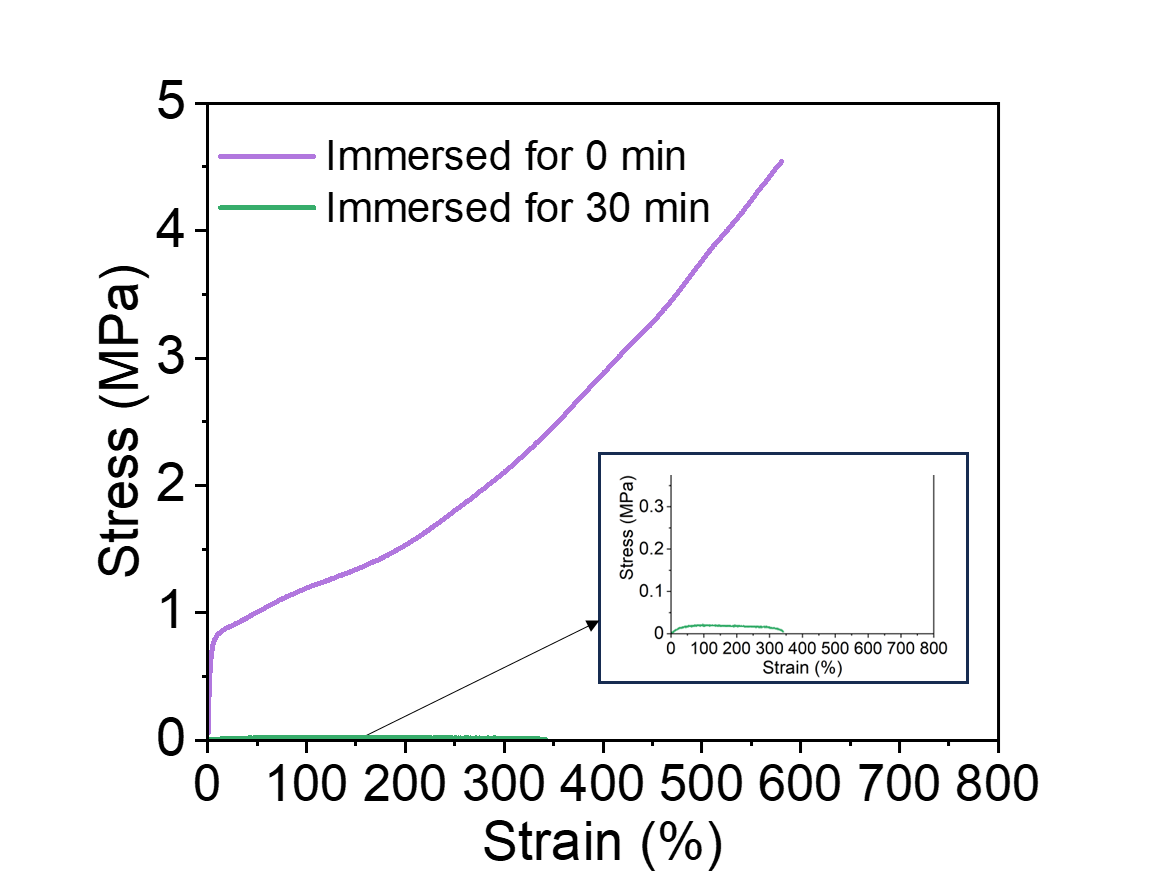


**Figure S3.** Stress-strain curves of hydrogel P (ACMO-*co*-AAm_7_) (2eq ChCl) after water immersion for 0 and 30 min.


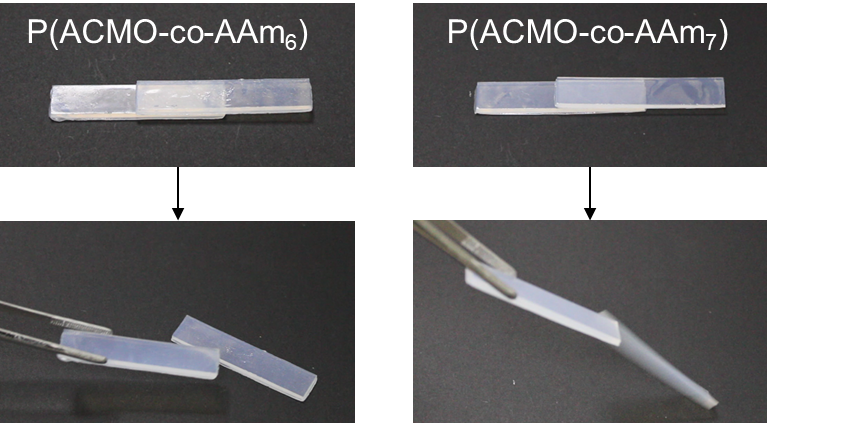


**Figure S4.** Photographs of the hydrogel P(ACMO-co-AAm_6_) and P(ACMO-co-AAm_7_) showing no adhesion in air.


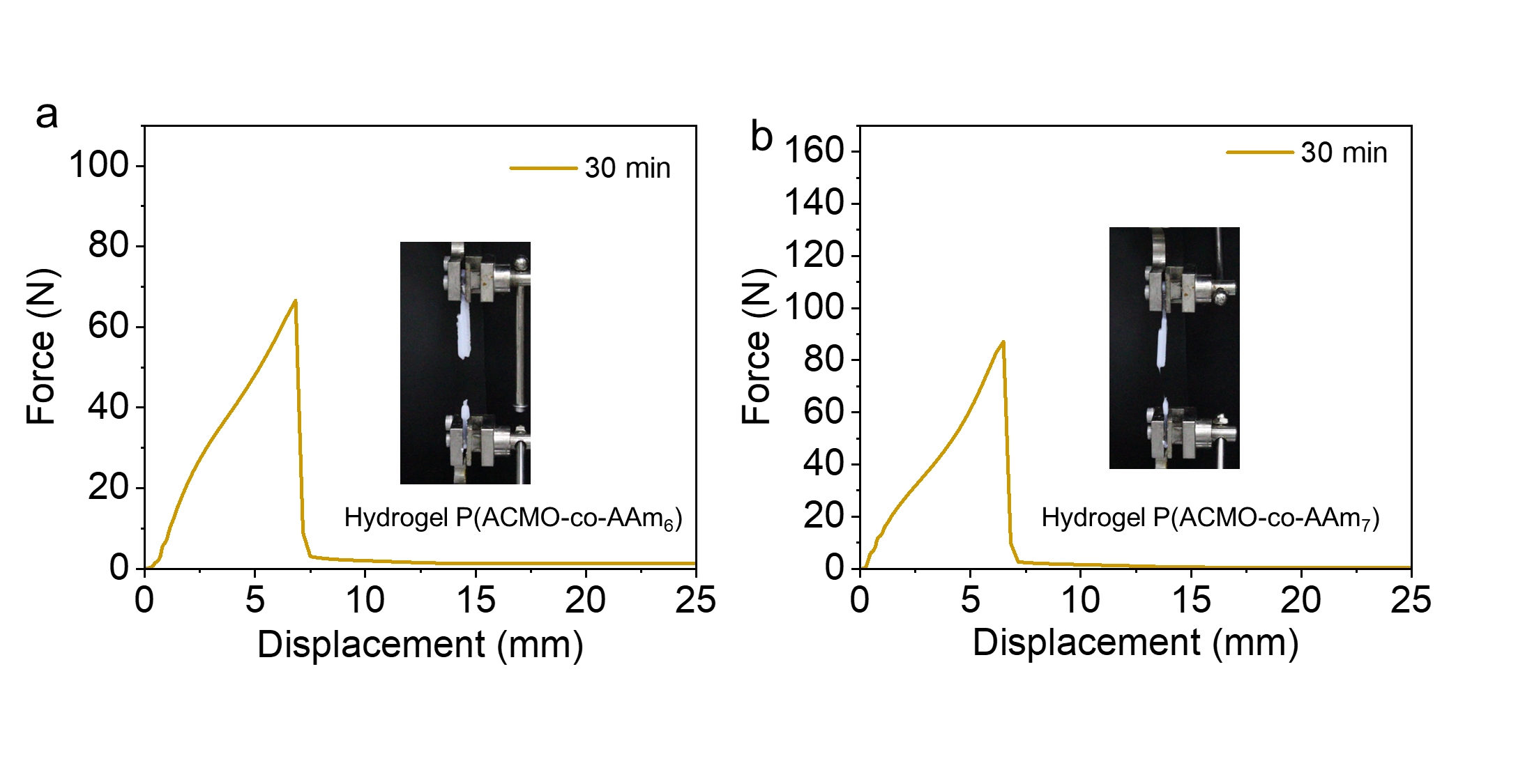


**Figure S5.** Force-displacement curves and photograph of (a) hydrogel P(ACMO-*co*-AAm_6_) and (b)P(ACMO-*co*-AAm_7_) after water immersion for 30 min.


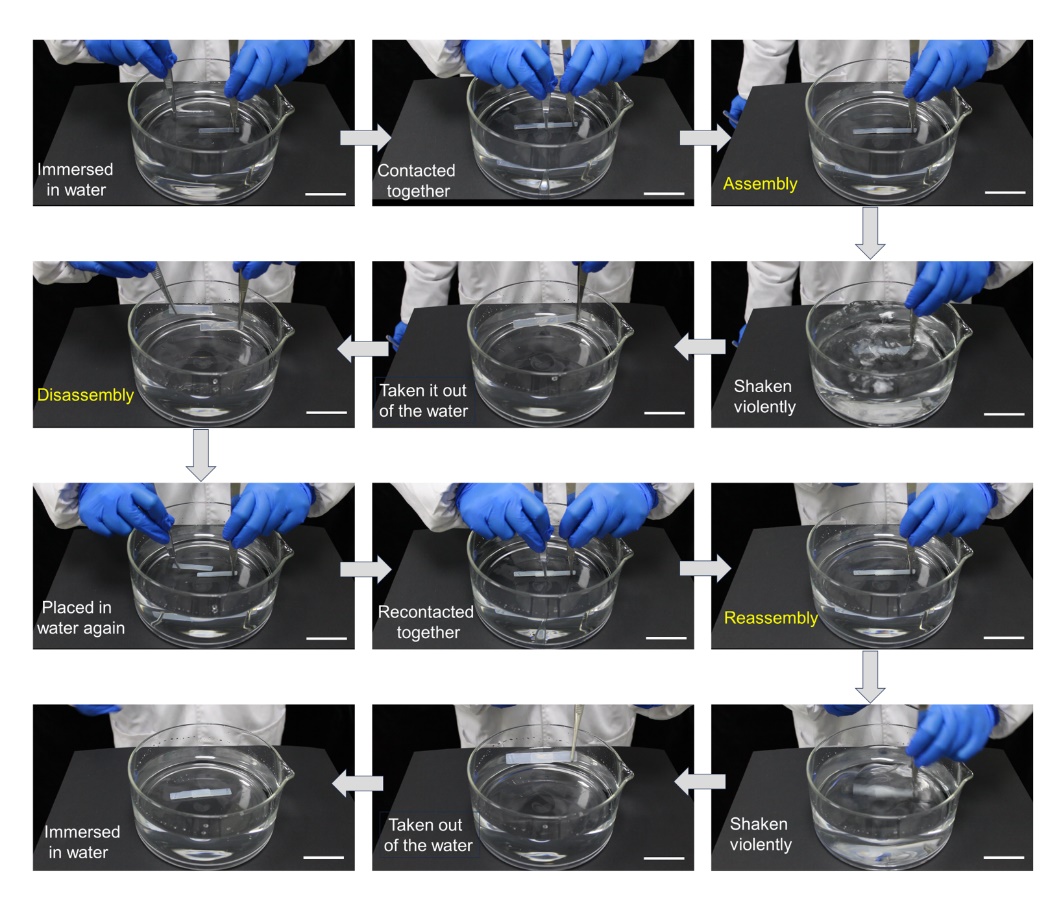


**Figure S6.** Photographs indicating underwater repeatable adhesion process of hydrogel P(ACMO-*co*-AAm_7_) modules. The scale bars are 5 cm.


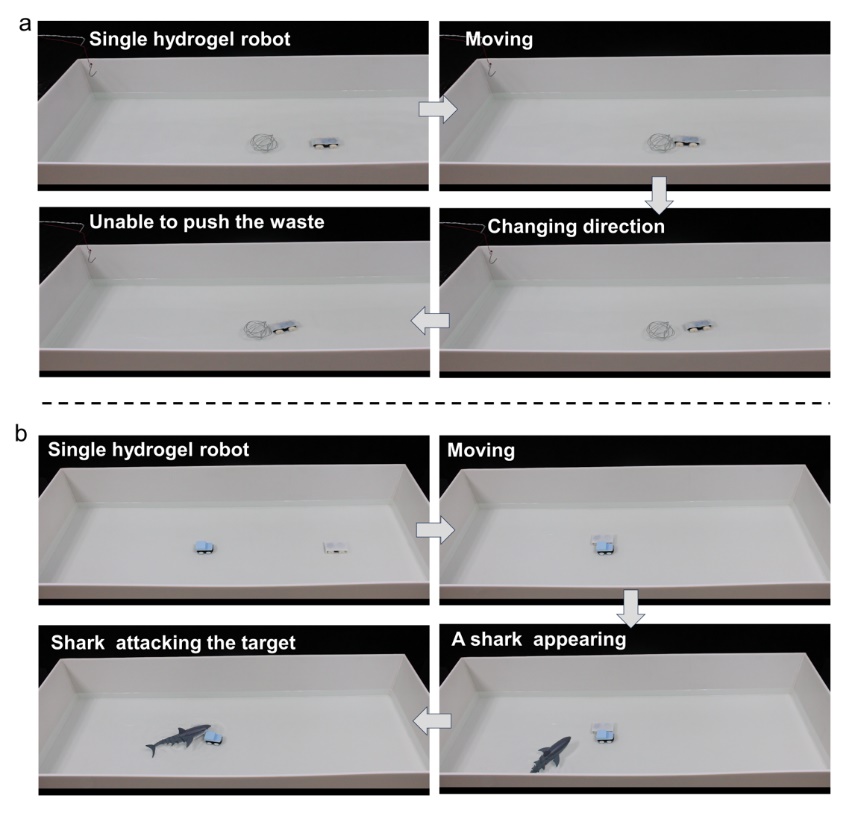


**Figure S7.** Photographs of (a) underwater waste removal task and (b) underwater rescue task performed by single hydrogel robot.


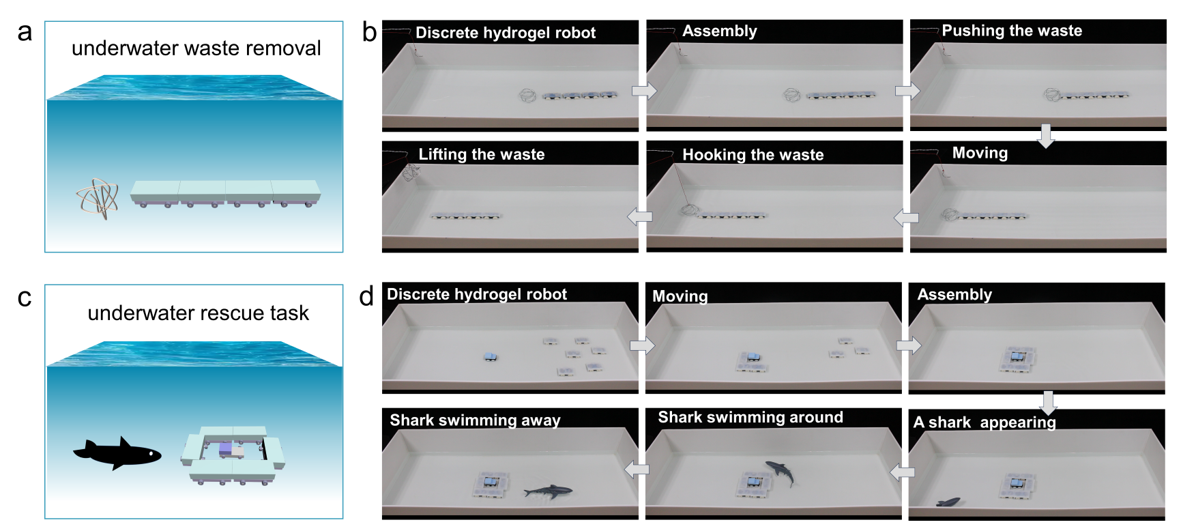


**Figure S8.** (a) Cartoon illustration of modular assembled hydrogel robots for underwater waste removal task performed by MAHR. (b) Photographs of MAHR in the process of performing underwater waste removal task. (c) Cartoon illustration of modular assembled hydrogel robots for underwater rescue task. (d) Photographs of MAHR in the process of performing underwater rescue task.

In order to test the underwater handling capability of hydrogel robot, an underwater waste removal scenario was simulated for comparative experiment of single hydrogel robot as well as MAHR. Firstly, a single hydrogel robot was placed on the right side of the underwater platform while the metal cage representing underwater waste to be cleaned was placed in the center. It was expected that the waste to be cleaned would be pushed to the designated position. For the single underwater robot, it is not possible to push the waste to move due to lack of power. Subsequently, four hydrogel robots were placed discretely on the right side of the underwater platform and assembled into a linear MAHR through interfacial hydrogen bonding. Once assembled, they were augmented with propulsive force to smoothly push the waste to the designated location, and the waste was eventually removed from water by a hook. Therefore, MAHR showed more powerful propulsion than single hydrogel robot in scenario-based tasks such as underwater transportation of heavy loads. Collaboration is a major feature of modular robot to accomplish tasks that cannot be completed by a single robot. In order to test the collaboration ability of hydrogel robot to cooperate with each other, we simulated an underwater rescue scenario for single underwater robot and MAHR respectively. A single underwater robot was placed on the right side of the underwater platform and the target to be rescued was placed in the center of the water. After single underwater robot moved to the target, an aggressive shark appeared, which easily detected the target and attacked it to knock it apart. Subsequently, six discrete hydrogel robots were distributed on the right side of the underwater platform, and by controlling them to move around the target separately, the six hydrogel robots were assembled into a square MAHR through interfacial adhesion and surrounded the target to protect it. When the shark appeared again, it failed to attack the target but just swam away. As a result, MAHR owned better functional flexibility to cope with complex environments and tasks through morphological reconfiguration compared to single underwater robot.
